# Fundamental insights on when social network data are most critical for conservation planning

**DOI:** 10.1111/cobi.13500

**Published:** 2020-09-05

**Authors:** Jonathan R. Rhodes, Angela M. Guerrero, Örjan Bodin, Iadine Chadès

**Affiliations:** ^1^ School of Earth and Environmental Sciences The University of Queensland Brisbane QLD 4072 Australia; ^2^ ARC Centre of Excellence for Environmental Decisions The University of Queensland Brisbane QLD 4072 Australia; ^3^ Centre for Biodiversity and Conservation Science The University of Queensland Brisbane QLD 4072 Australia; ^4^ School of Biological Sciences The University of Queensland Brisbane QLD 4072 Australia; ^5^ Stockholm Resilience Centre Stockholm University Stockholm SE‐106 91 Sweden; ^6^ CSIRO Ecosciences Precinct Dutton Park QLD 4102 Australia

**Keywords:** artificial intelligence, social network analysis, species distributions, stochastic dynamic programing, value of information, análisis de redes sociales, distribución de las especies, inteligencia artificial, programación estocástica dinámica, valor de la información, 人工智能, 社会网络分析, 物种分布, 随机动态规划, 信息价值

## Abstract

As declines in biodiversity accelerate, there is an urgent imperative to ensure that every dollar spent on conservation counts toward species protection. Systematic conservation planning is a widely used approach to achieve this, but there is growing concern that it must better integrate the human social dimensions of conservation to be effective. Yet, fundamental insights about when social data are most critical to inform conservation planning decisions are lacking. To address this problem, we derived novel principles to guide strategic investment in social network information for systematic conservation planning. We considered the common conservation problem of identifying which social actors, in a social network, to engage with to incentivize conservation behavior that maximizes the number of species protected. We used simulations of social networks and species distributed across network nodes to identify the optimal state‐dependent strategies and the value of social network information. We did this for a range of motif network structures and species distributions and applied the approach to a small‐scale fishery in Kenya. The value of social network information depended strongly on both the distribution of species and social network structure. When species distributions were highly nested (i.e., when species‐poor sites are subsets of species‐rich sites), the value of social network information was almost always low. This suggests that information on how species are distributed across a network is critical for determining whether to invest in collecting social network data. In contrast, the value of social network information was greatest when social networks were highly centralized. Results for the small‐scale fishery were consistent with the simulations. Our results suggest that strategic collection of social network data should be prioritized when species distributions are un‐nested and when social networks are likely to be centralized.

## Abstract 2

*Article impact statement*: The value of collecting social network information for conservation planning depends on both species distributions and social network structure.

## Abstract (es)

Ideas Fundamentales sobre Cuándo Son Más Importantes los Datos de las Redes Sociales para la Planeación de la Conservación

**Resumen**

Conforme se aceleran las declinaciones de la biodiversidad, existe una exigencia urgente para asegurar que cada dólar que se gasta en conservación contribuya a la protección de las especies. La planeación sistemática de la conservación es una estrategia usada extensivamente para lograr esto, aunque cada vez existe una mayor preocupación por que integre las dimensiones sociales humanas de la conservación para que sea una estrategia efectiva. Aun así, es insuficiente el conocimiento fundamental sobre cuándo son más importantes los datos sociales para orientar a las decisiones de planeación de la conservación. Para tratar con este problema identificamos los principios novedosos que sirven como guía para la inversión estratégica en la información de las redes sociales para la planeación sistemática de la conservación. Consideramos un problema común para la conservación; identificar con cuáles actores sociales, dentro de una red social, interactuar para incentivar el comportamiento de conservación que maximice el número de especies protegidas. Usamos simuladores de redes sociales y de especies distribuidas a lo largo de nodos de redes para identificar las estrategias dependientes del estado más convenientes y el valor de la información provenientes de las redes sociales. Hicimos lo anterior para una gama de estructuras de redes de motivos y distribución de especies y aplicamos la estrategia a una pesquería a pequeña escala en Kenia. El valor de la información proveniente de las redes sociales depende firmemente tanto de la distribución de las especies como de la estructura de la red social. Cuando las distribuciones de las especies se encontraban extremadamente anidadas (es decir, cuando los sitios pobres en cuanto a cantidad de especies son subconjuntos de sitios ricos en cantidad de especies), el valor de la información proveniente de las redes sociales casi siempre fue bajo. Esto sugiere que la información sobre cómo se distribuyen las especies en una comunidad es crítica para determinar si invertir o no en la recolección de datos provenientes de las redes sociales. Como contraste, el valor de este tipo de información fue mucho mayor cuando las redes sociales estaban sumamente centralizadas. Los resultados de la pesquería a pequeña escala fueron compatibles con las simulaciones. Nuestros resultados sugieren que la recolección estratégica de datos a partir de las redes sociales debería ser prioridad cuando las distribuciones de las especies no se encuentran anidadas y cuando sea probable que las redes sociales estén centralizadas.

## 摘要

随着生物多样性丧失不断加剧, 人们迫切需要确保保护投资都能有效用于物种保护。系统保护规划是实现这一目标的一种广泛使用的方法, 但人们越来越多地认为, 系统保护规划必须更好地结合保护中的人类社会维度才能发挥作用。然而, 目前还缺乏关于社会数据何时对保护规划决策最重要的基本见解。为此, 我们提出了新的原则来指导系统保护规划中社会网络信息的战略投资。本研究分析了一个常见的保护问题, 即在一个社会网络中应如何确定参与保护的社会行动者, 以激励能够保护最多数量物种的保护行动。我们模拟了社会网络和分布在网络节点上的物种来确定基于状态的最优策略和社会网络信息价值。接下来, 我们对一系列网络模体结构和物种分布进行了分析, 并将这种方法应用于肯尼亚的一个小型渔场。社会网络信息价值与物种分布和社会网络结构密切相关。当物种分布高度嵌套时 (即物种贫乏位点是物种丰富位点的子集), 社会网络信息价值几乎总是很低。这表明, 关于物种在网络中分布的信息对于决定是否投资于社会网络数据收集十分重要。相反, 当社会网络高度集中时其信息价值最大。小型渔场的结果与模拟结果一致。我们的研究结果表明, 当物种非嵌套分布且社会网络可能很集中时, 应该优先考虑战略性收集社会网络数据。**【翻译: 胡怡思; 审校: 聂永刚】**

## Introduction

As Earth's biodiversity‐loss crisis grows, efficient and effective investment of scarce conservation resources to protect species is vital (Barnosky et al. 2011). Systematic conservation planning aims to achieve this by formally identifying the actions that most cost‐effectively protect species and is used widely (McIntosh et al. 2017). There is, however, a growing call for conservation planning to better integrate human social dimensions in planning processes to ensure successful outcomes (Ban et al. 2013; Bennett et al. 2017*b*). Achieving this will require much greater investment in the collection of social data. Yet, currently little is known about when collection of social data is most valuable for conservation planning and when it is least valuable. Further, there is considerable interest in understanding priorities for ecological data collection through value of information analysis (Bal et al. 2018; Nicol et al. 2019), but this has not been extended to social data.

Information on the social dimensions of conservation problems can be beneficial for a wide range of reasons. These include describing the social context, diagnosing likely success of conservation activities, and characterizing social values to inform objective setting for decision making (Bennett et al. 2017*a*). Identifying how social systems influence participation in conservation interventions, such as land purchases, payments for ecosystem services, or co‐management is a key role for the social sciences. Participation in a conservation intervention will depend on the intrinsic propensity of people to do so (Guerrero et al. 2010), but can also be influenced by interactions among people (Díaz‐José et al. 2016; Dobson et al. 2019). In particular, achieving widespread uptake of conservation interventions can be strongly influenced by the nature of the networks of connections among people or organizations (Alexander et al. 2018). When these network connections are important for maximizing the success of conservation interventions, social network information and analysis (Borgatti et al. 2009) could be key to designing effective conservation programs. Consequently, there is growing interest in the use of social network data in conservation decision making (Mills et al. 2014; Guerrero et al. 2020).

Social network analysis has been used to explain a wide range of social phenomena, such as collaboration, innovation, social influence, diffusion of behavior, and access to new knowledge (Valente 1996; Borgatti et al. 2009; Aral & Walker 2012; Huggins et al. 2012). It has also been used to demonstrate that the characteristics of social networks can be a key determinant of the best choice of strategic interventions to achieve a desired outcome (Valente 2012). In natural resource management, it has been applied to understand management success and to show that priority interventions depend on the structure and nature of social networks (Barnes et al. 2016; Bodin 2017; Matous & Wang 2019). In the conservation literature, social network analysis is an emerging approach for integrating social data into conservation planning (Mills et al. 2014; Guerrero et al. 2020). For example, it has been used in spatial conservation planning to identify areas where actors are highly connected because that is where conservation interventions should be most successful (Mills et al. 2014). Yet, there is little understanding of the key factors that determine when it is valuable to invest conservation resources in collecting new social network information and when it is not for conservation planning. This understanding is critical if the benefits of social data for biodiversity conservation are to be maximized.

We used well‐known dynamic network models of spread of human behavior (Cointet & Roth 2007; Kleinberg 2007), dynamic optimization (Hoey et al. 1999; Costello & Polasky 2004), and value of information analysis to provide insights into the value of social network data for conservation planning. We developed a new conservation planning approach that links models of social network dynamics with data on species distributions to optimize which social actors to engage with to conserve species. We then used this approach, combined with simulations, to identify key characteristics of social network and species distributions that determine the value of social network information in identifying optimal strategies. Finally, we applied our approach to a real system based on data from a small‐scale fishery in Kenya.

## Methods

### Conservation Problem Formulation

To formulate the conservation planning problem, we started with a social network in which each node consisted of an individual or organization (hereafter an actor) and a spatial area containing species that each actor has exclusive management rights over (hereafter site). This characterizes a wide range of conservation planning problems, such as conservation on private land, where landholders have exclusive land‐use rights to their properties (Kamal et al. 2015), or in marine systems, where fisheries have exclusive access to fishing zones, such as in territorial user rights fisheries (TURFS) (White & Costello 2011). Typical actor‐level conservation interventions to achieve species protection in these cases include attempts to purchase properties, incentive payments to landholders, or investment in co‐management of fishing zones. We assumed that a decision maker (e.g., conservation organization or government department) aims to optimally choose actor‐level interventions over time to maximize the number of species protected.

### Social Network Dynamics and Network Structures

**figure 1:**
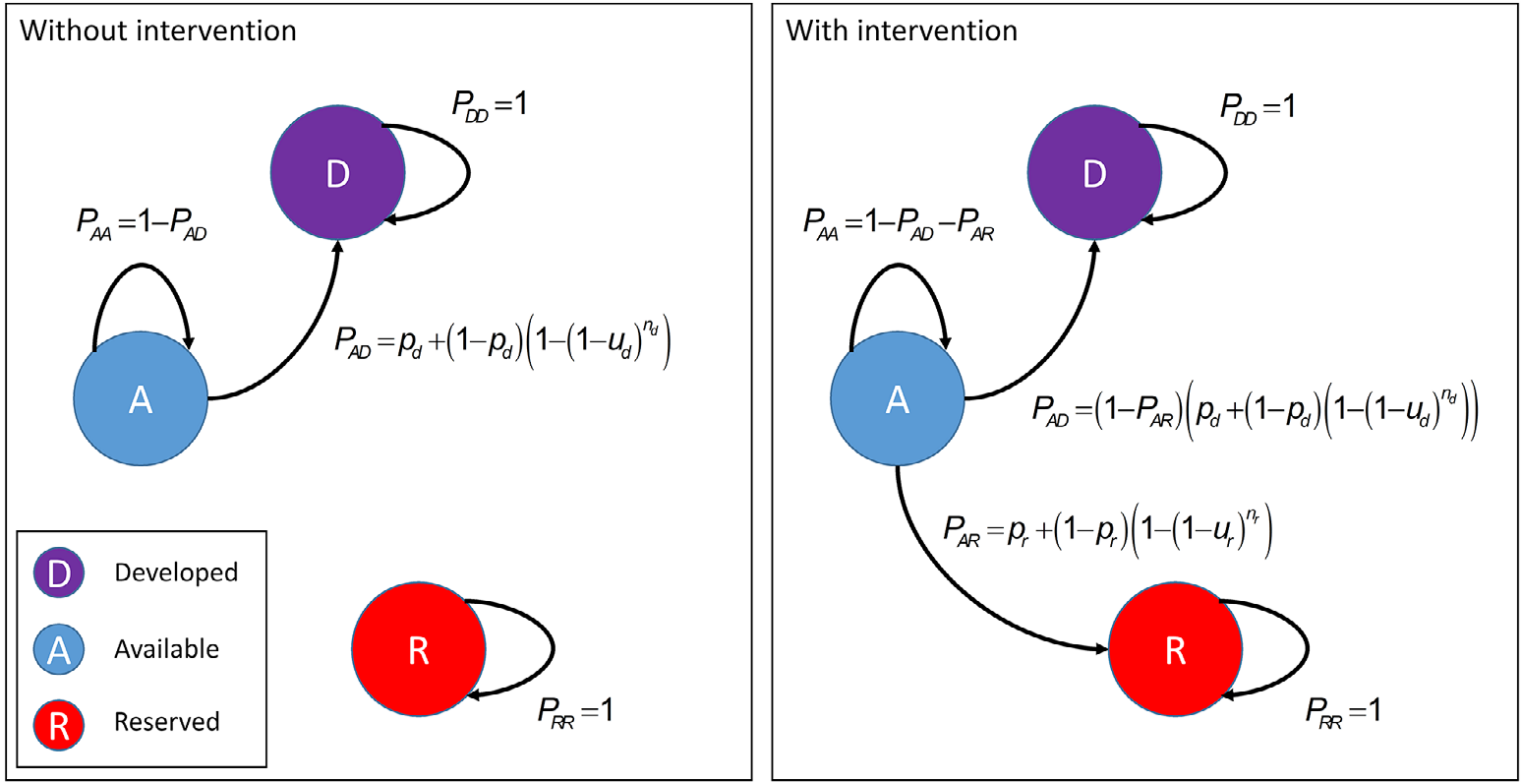
The specification of transition probabilities for a network node (i.e., actor and associated site) without and with a conservation intervention (P_i_, transition probability from state i to state j for the available [A], developed [D], and reserved [R] states; p_d_, probability, independent of the network, that an actor associated with a site decides to develop the site; p_r_, probability, independent of the network, that an actor associated with a site decides to reserve the site; u_d_, probability that an actor, who has already developed a site, influences another actor to develop, given they are connected in the network; u_r_, probability that an actor, who has already reserved a site influences another actor to reserve, given they are connected in the network; n_d_, number of actors who have already developed their sites that the actor is connected to; n_r_, number of actors who have already reserved their sites that the actor is connected to). In network terminology, parameters u_d_ and u_r_ are measures of link strength.

We assumed that each site and associated actor can be in 1 of 3 states (available, developed, or reserved) and that stochastic transitions between these states depend on the actors’ behaviors (Fig. 1). From the available state, actors can choose to do nothing (in which case the associated site stays in the available state), develop the associated site, or designate the associate site as a reserve. The probability of an actor making each of these decisions depends on whether an intervention has targeted the actor and on influence on the actor through the social network (Fig. 1). We assumed network links represent pathways for social influence and information sharing, so that actors connected to each other tend to behave more similarly than those who are not connected (Fig. 1 & Supporting Information). This assumption aims to replicate evidence for the spread of behaviors through social networks (Aral & Walker 2012; Contractor & DeChurch 2014) that occur via processes, such as the diffusion of knowledge and innovation (Bandura 2001; Rogers 2003), direct influence (Bond et al. 2012), and spread of social norms (Fowler & Christakis 2010). These phenomena are important for the spread and adoption of sustainable management and land‐use practices (Abrahamse & Steg 2013). A transition to the reserved state is only possible when an actor has been targeted by an intervention. This is because we assumed there is a tendency for available nodes to transition only to the developed state unless the necessary conditions are in place to facilitate, or incentivize, transition to the reserved state (i.e., *p_r_* = 0 and *u_r_* = 0 in Fig. 1 when there is no intervention, where *p_r_* is the probability an actor decides to reserve the site, independent of the network, and *u_r_* is the probability an actor who has reserved their site influences another actor to reserve). This mimics the aim of many interventions to achieve species protection, or sustainable natural resource use, such as payments for ecosystem services, land purchases, fishery co‐management interventions, and technical assistance to enhance the spread of favorable practices (Evans et al. 2011; van Straalen & Korthals Altes 2014; Naeem et al. 2015; Mascia & Mills 2018). Once in the reserved or developed states, no further transitions are possible (i.e., they are absorbing states). Our model of networks dynamics (Supporting Information) is similar to cascade models of behavior spread and epidemic models of disease spread (susceptible‐infected models), which provide realistic patterns of social influence or behavior spread (Cointet & Roth 2007; Kleinberg 2007). The one difference being that we assumed the spread of the reserved state was only possible if accompanied by an intervention.

**figure 2:**
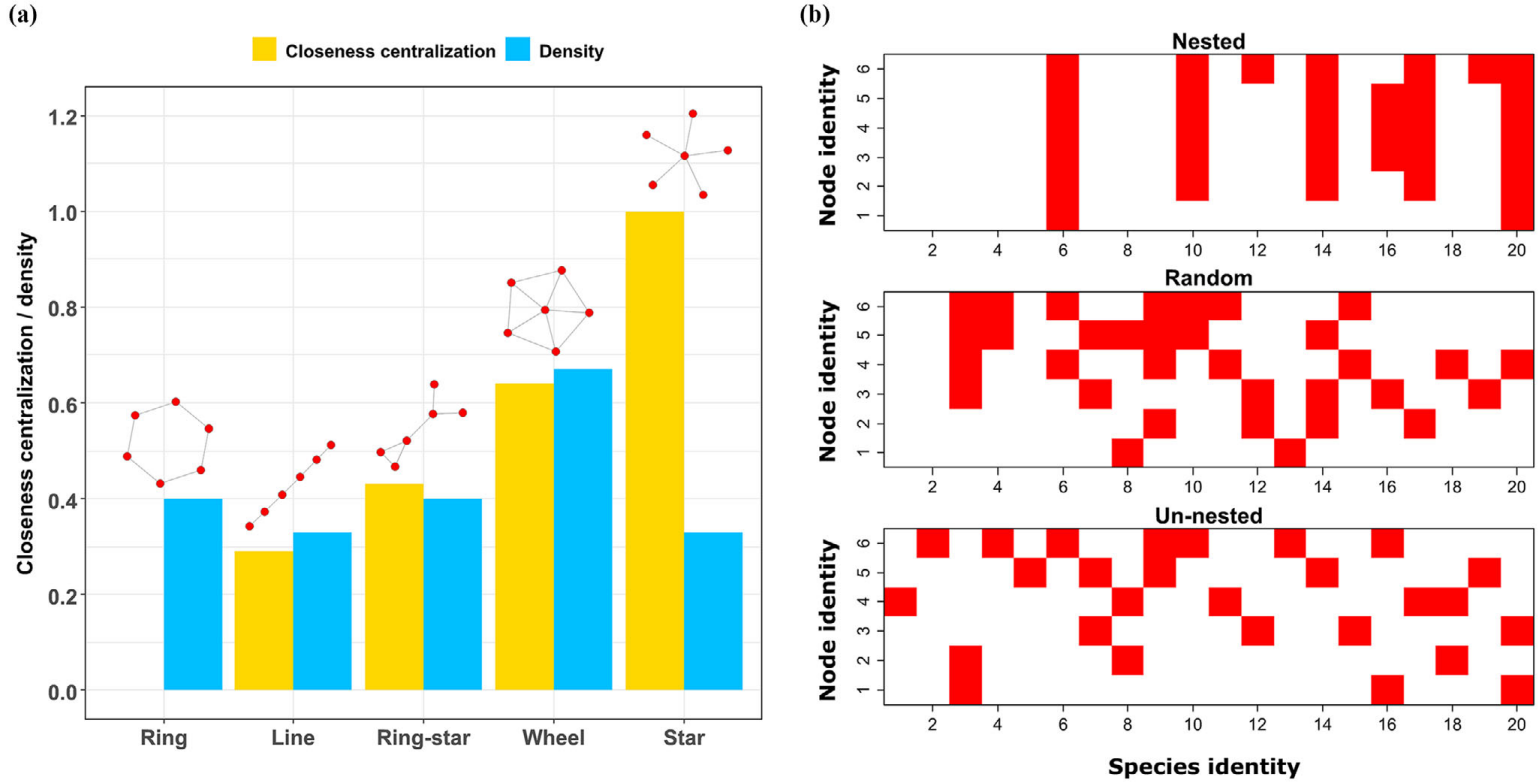
(a) Network motif structures considered and their network closeness centralizations (measure of likelihood that 1 node has high closeness centrality relative to other nodes) and network densities (measure of number of links in the network relative to number of possible links). Closeness centrality for a node is a measure of the distance between the node and all other nodes (high closeness centrality indicates short distances). (b) Example of simulated species distributions across nodes for different levels of nestedness (squares, which species are present in which nodes; nested, species allocated to nodes to achieve a nearly perfectly nested distribution; random, species allocated to nodes to achieve a completely spatially random distribution; un‐nested, species allocated to nodes to avoid nestedness).

We considered a number of 6‐node motif (Milo et al. 2002; Chadès et al. 2011) network structures (Fig. 2). These network structures (ring, line, ring‐star, wheel, and star) were chosen to represent small networks along a gradient of closeness centralization. Although these small networks do not represent specific cases of real networks, they are likely to represent motifs that exist in real social systems. For example, the star motif is likely common in systems where there is a single highly influential landholder (Knight et al. 2010), and the line motif may appear in linearly connected systems, such as TURFS arranged along a linear coastline (White & Costello 2011). Network closeness centralization (a measure of the variation in the closeness of each node to all other nodes) was used as a measure of the ability of a small number of specific nodes to cause behavior spread to many other nodes due to their high connectivity (Mbaru & Barnes 2017). We, therefore, hypothesized that network closeness centralization would be a key determinant of the sequence and number of interventions required. All networks had similar network density (number of links relative to number of possible links), except for the wheel network, which had network density approximately 50% higher than the other networks (Fig. 2).

### Species Distributions and Extinction Dynamics

We assumed that species were distributed randomly across sites, but that their distribution was structured across nodes based on species nestedness (Supporting Information). Species nestedness is the extent to which species present at sites are nested subsets of more species rich sites and is an important determinant of the number of sites needed to represent species in protected areas (Cutler 1994; Fischer & Lindenmayer 2005). We, therefore, hypothesized that species nestedness is an important determinant of the number and sequence of interventions and so likely to influence the value of social network information. We assumed that species go extinct at a site under the developed state, but persist under the available and reserved states. However, extinction from the available state can still occur through a subsequent transition to the developed state.

### Solving for the Optimal Sequence of Interventions

We assumed discrete time steps and that, at each time step, the decision maker can target a single actor for an intervention to incentivize, or facilitate, the reserve behavior. These interventions influence the transition probabilities for the targeted actor according to the equations in Fig. 1. As such, an intervention can result in the protection of species directly at the targeted site or actor, but also indirectly by facilitating the spread of reserve behaviors or constraining the spread of development behaviors across the network. We solved the decision problem as a factored Markov decision process (Hoey et al. 1999) with stochastic dynamic programing to identify the optimal state‐dependent interventions that maximize the expected time‐discounted sum of the number of species represented in reserves over an infinite time horizon, less a notional cost for each intervention (Supporting Information). Although we assumed an infinite time horizon and only reserved sites contribute to the benefit function, we recognize that a wide range of other benefit functions and time horizons could be considered.

### Simulations and Calculating the Value of Social Network Information

To calculate the value of social network information, we simulated outcomes for the optimal sequence of interventions, first assuming that perfect information about the social network is available to the decision maker and then assuming that social network information is not available. The value of social network information was then calculated as the difference in conservation outcomes (Supporting Information) when decisions are made with and without social network information. We calculated the value of social network information for each of our 6 motifs for a pool of 20 species: probability an actor, independent of the network, decides to reserve (*p_r_*) was assumed to be 0.2; probability an actor, independent of the network, decides to develop (*p_d_*) was assumed to be 0.2 (Fig. 1). We ran simulations across the full range of species nestedness values (*φ* = {0.1, 0.2, 0.3, 0.4, 0.5, 0.6, 0.7, 0.8, 0.9, 1.0}), values for reservation‐link strength (*u_r_* = {0.0, 0.1, 0.2, 0.3, 0.4, 0.5, 0.6, 0.7, 0.8, 0.9, 1.0}), and values of development‐link strength (*u_d_* = {0.0, 0.1, 0.2, 0.3, 0.4, 0.5, 0.6, 0.7, 0.8, 0.9, 1.0}) (Fig. 1). This resulted in 6,050 simulated instances per motif that we calculated the value of social network information for. We then quantified the association between the value of social network information, nestedness, and link strengths for each motif with generalized linear mixed models (Supporting Information).

### Application to a Small‐Scale Fishery

We applied our approach to a small‐scale fishery in Kenya consisting of fisher groups using different gear types (speargun, handline, seine net, ring net, and gillnet) that fish in wholly, or partly, spatially separated fishing sites, but where fisher groups are connected through a social network (Supporting Information) (Crona & Bodin 2006; Bodin et al. 2014). We made the reasonable simplifying assumption that each group and associated fishing site were separate nodes within a network because of their spatial separation. The fisher groups in the system share information through the social network, so knowledge and information flow provides a potential mechanism for the spread of behaviors. The gear types each fisher group uses are associated with specific fish species harvested in each fishing site. The social network (including the relative strength of links) and species associated with each fisher group are described in Bodin et al. (2014).

The fishery is considered to be on an unsustainable trajectory in the long term (McClanahan et al. 1997; Ochiewo 2004). Therefore, we considered potential interventions with fisher groups to facilitate, or incentivize, them to not transition to unsustainable harvesting that could result in the local loss of species. The specific types of intervention could include poverty alleviation and co‐management, which are commonly used as management interventions in small‐scale fisheries (Evans et al. 2011; Mills et al. 2011). We constructed the social network for the fishery to include heterogeneity in the strengths of social links between fisher groups (Crona & Bodin 2006; Bodin et al. 2014), which was in contrast to the simulated networks, where we assumed that link strengths were homogenous. We then identified the optimal series of interventions in the network and determined the value of social network information across a range of assumptions about link strengths. We contrasted the value of social network information for this real system with the simulated networks.

## Results

**figure 3:**
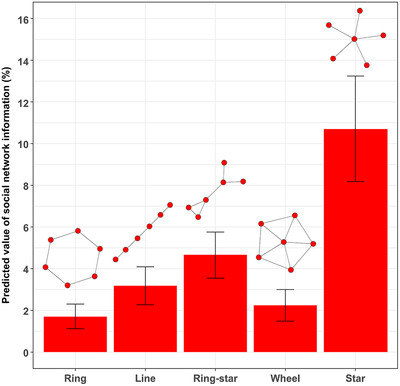
The value of including social network information in the optimization of conservation interventions for each network motif. The value of social network information represents the expected percent improvement in conservation benefits achieved by including social network information in the prioritization (error bars, 95% credible intervals; values, predictions from generalized linear mixed‐effects models fitted to the simulated data [with other parameters set at mean values]).

On average, the incorporation of social network information improved conservation outcomes by 1.7–10.7% (Fig. 3). There was considerable variation across the different network structures but, in general, the value of social network information increased with network closeness centralization. The value of social network information for the star network (the most centralized) was significantly higher than for all other networks (*p* <0.05). The exception to this pattern was the wheel network. Although this network has high centralization, it also has much higher network density than the other networks (Fig. 2). High network density increases the number of possible pathways for the spread of behaviors across the whole network and so likely reduces the benefit of targeting particular parts of the network through strategic interventions. This consequently reduces the benefit of having information about the specific structure of the social network for targeting conservation interventions.

When social network information was ignored, the optimal decision was always to first target the most species‐rich site. However, when social network information was considered, the most species‐rich site was less likely to be targeted first, and this depended on the network. For example, for the star network, the most species‐rich site was targeted first only 66% of the time across all simulations, whereas for the ring network, the most species‐rich site was targeted first as much as 92% of the time. For the star network, the most central node was not necessarily the first node targeted (targeted only 62% of the time) because the optimal decision also depended on the distribution of species. However, for all networks, the most centralized node was targeted first more often when social network information was included, than when social network information was ignored. This emphasis on targeting central nodes when social network data are available appeared to be one mechanism driving the positive value of social network information.

**figure 4:**
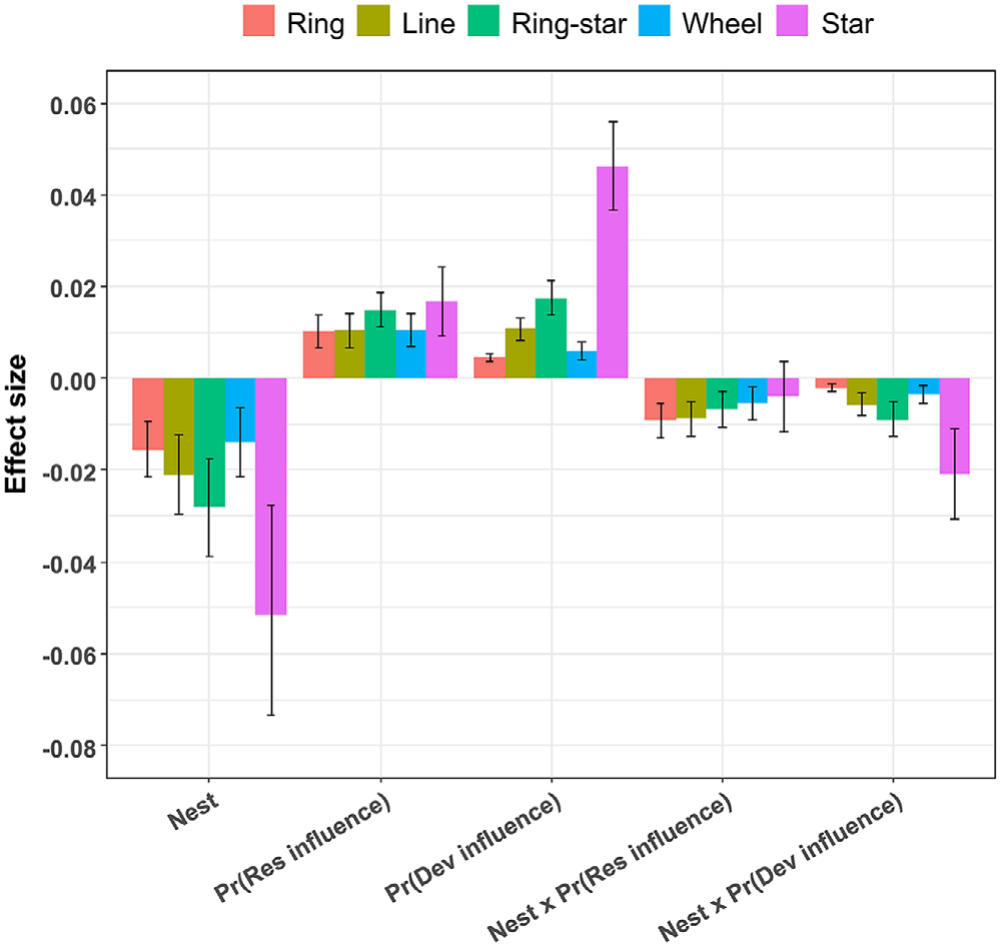
Effect of species nestedness, reserve influence probability, development influence probability, interaction between species nestedness and reserve influence probability, and interaction between species nestedness and development influence probability on the value of social network information(Nest, nestedness; Pr(Res influence), reserve influence probability; Pr(Dev influence), development influence probability; x, an interaction). Values are predictions from the generalized linear mixed‐effects models fitted to the simulated data.

Increasing species nestedness significantly reduced the value of social network information for all networks (*p* <0.05), and this effect tended to be highest for the most centralized networks (Fig. 4). In contrast, increasing the probability of reserve and development influence (i.e., link strength) increased the value of information for all networks (*p* <0.05), and this effect was generally highest for the most centralized networks (Fig. 4). The effects of the interaction between species nestedness and influence probabilities were generally negative (*p* <0.05), indicating that, as species nestedness increased, influence probability became less important for determining the value of information (Fig. 4). The effects of network structure, influence probabilities, and species nestedness resulted in substantial differences in the value of network information across these parameters (Supporting Information). In particular, the value of information for the ring, line, and wheel networks was all low (<10%), regardless of other parameter values. However, the value of information for the star network was considerable (> 25–35%) when species distributions were un‐nested and influence probabilities were high. Yet, even for the star network, the value of information was low (<10%) when species distributions were nested, regardless of influence probabilities.

**figure 5:**
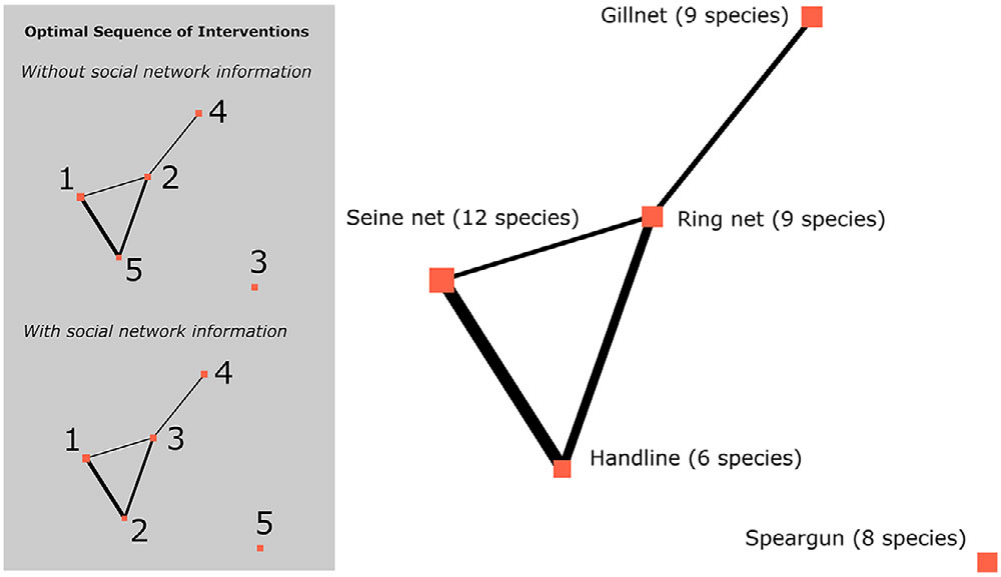
Small‐scale fishery social network and optimal sequence of conservation interventions with and without social network information. Size of nodes are proportional to the number of species and line widths are proportional to the link strengths (influence probabilities).

The small‐scale fishery network had intermediate network closeness centralization (0.29) and network density (0.4), but had one disconnected node (Fig. 5). Species nestedness was not significantly different from random based on Brualdi and Sanderson's (1999) discrepancy index (discrepancy statistic = 18, *p* = 0.91). That is, species were not strongly nested or strongly un‐nested. However, the value of social network information was relatively low; improvement in fish species protection was on average only 2.6% with social network information. This was true even when influence probabilities were assumed to be high.

Prioritizations with and without social network information were similar. With social network information, the optimal strategy was to first intervene at the most species‐rich node (seine net group), followed by the handline group (Fig. 5). The strong link between the seine net and handline groups increased the probability of a successful subsequent intervention for the handline group if a successful intervention for the seine net group had already been implemented. This outweighed the expected benefits of intervening at a more species‐rich node in the second step. Without social network information, the optimal strategy was again to first intervene at the seine net group, but then to intervene at the ring net group (second most species‐rich site), rather than the handline group (Fig. 5). The isolated speargun group was relatively highly prioritized when the social network was ignored, but was of low priority when the social network was accounted for (Fig. 5). These differences in optimal interventions resulted in some benefit of social network information. Yet, only moderate network centralization, combined with the random distributions of species across nodes (as opposed to being un‐nested), meant the benefit was relatively low.

## Discussion

We found that the conservation benefit of social network information for conservation planning depends strongly on the interaction between social network structure and the distribution of species. In general, the value of knowing the structure of highly centralized networks was higher than knowing the structure of less centralized networks. Nevertheless, under some ecological conditions, we discovered that the value of knowing the social network was always low. This occurred when species distributions were highly nested (i.e., when conservation values were concentrated in only one or a few sites). In this case, only a small proportion of sites needed to be reserved to protect most species, so the optimal strategy was predominantly driven by where the most species rich sites were. In contrast, when species distributions were un‐nested (i.e., when conservation values were more evenly spread across sites), information on the social network became more strategically important for decision making, particularly for highly centralized networks. In this case, a larger proportion of sites needed to be reserved to protect all species, so the influence of the network for achieving this became more important.

The implications of heterogeneity in species distributions for the value of information on the social dimensions of conservation have been suggested previously (Cowling et al. 2010), but have not been formally demonstrated until now. Governments and organizations commonly make strategic‐planning decisions to influence, or incentivize, social, institutional, and administrative units that interact with each other to preserve biodiversity (Robinson et al. 2016). Our results indicate that conservation organizations that already have biodiversity data could use that information to help determine whether to strategically invest in collecting detailed social network information. Although we did not explicitly consider heterogeneity in the social characteristics of the actors, we hypothesize that high actor heterogeneity may also tend to reduce the value of social network information. We found that, despite high heterogeneity in link strengths in the fishery case study, results were still consistent with the simulations, but the broader implication of heterogeneity in link strength on the value of social network information is unknown. These are important questions for future research and additional factors for decision makers to consider.

The structure of social networks can drive the dynamics of social or governance systems and consequently influence environmental outcomes (Bodin 2017). The dynamics of our networks mimic the spread of social behaviors, so that connected actors are more likely to behave similarly, as would be expected with the diffusion of knowledge and ideas in collaborative networks. High network densities tend to generate rapid spread of behaviors across the entire network. However, high levels of centralization tend to result in particular actors, or clusters of actors, being responsible for generating the rapid spread of behaviors. From a strategic intervention perspective, it can be critical to have information on where these influential actors are in the network. Identifying where these actors are is analogous to identifying well‐connected or influential people to advance environmental or conservation objectives (Knight et al. 2010). Our results suggest that, when social networks are thought, a priori, to be highly centralized, then characterizing the specific social network is likely to be key to achieving conservation success.

We designed our study to provide general mechanistic insights about the value of collecting social network information for conservation planning and provide optimal solutions to the problem of maximizing the number of species protected in social network motifs. We focused on motifs (Chadès et al. 2011) because this allowed us to generate exact optimal solutions for many thousands of replicates across different motifs and parameter combinations and identify key factors determining the value of social network information. Yet, many real‐world networks are much larger than we assumed in our simulations. Understanding whether principles derived from network motifs hold for larger networks is an active area of research (Benson et al. 2016). In our case, a key challenge to investigating larger networks is finding the optimal sequence of interventions for larger networks in which complexity increases exponentially with the number of nodes. Approaches, such as approximate dynamic programing, could be a way to find suboptimal solutions for much larger networks (Wang et al. 2015). Addressing these issues for larger networks is an important area for future research.

We assumed the distribution of species was known and asked questions about when information on the social network was most valuable. Of course, network structure and the distribution of species are often both uncertain. An equally important question, therefore, is, when is new information on the ecological system most valuable? Although some researchers have explored the value of ecological information for conservation (Maxwell et al. 2015; Bal et al. 2018), very little guidance is available in the context of social‐ecological systems. An important next step is, therefore, to consider the value of information on both social and ecological components, so that research effort can be prioritized across the full social‐ecological system (Davis et al. 2019). As conservation planning strives to better integrate information on social‐ecological systems, taking a more holistic social‐ecological approach to strategic prioritization of research and data collection to resolve uncertainties is vital.

Finding interventions in social‐ecological systems that achieve desired environmental outcomes is a central question in broad areas of environmental and natural resource management (Mbaru & Barnes 2017) and in many other fields, such as biosecurity, public health, and medicine (Smith & Christakis 2008; Kim et al. 2015; McAllister et al. 2015). Our approach, therefore, also has the potential for much broader application to problems that require actor‐level interventions in social networks. By incorporating alternative objective functions (e.g., social or economic objectives) and types of interventions, it could facilitate the finding of optimal strategies for a range of problems, such as interventions to limit spread of disease (Preciado et al. 2013). This may require a consideration of very different forms of spread models, such as those required to model spread based on threshold responses (Valente 1996), but incorporating these into our framework is entirely feasible.

Our results provide timely insights into the value of learning about social systems to improve the success of conservation planning. We found that the value of information about social networks that constrain and facilitate the spread of conservation behaviors depends strongly on the interaction between the distribution of species and network structure. There is an urgent need for more efficient and effective use of scarce conservation resources to reverse the current loss of biodiversity and meet global conservation goals. By deriving guidance on how to strategically prioritize social data collection, we have provided a new exciting pathway to maximize the effectiveness of conservation planning and many other related decision problems.

## Supporting information

Supplementary methods related to social network dynamics, species distributions, decision problem and optimization, value of social network information, small‐scale fishery (Appendix S1), supplementary figures and results (Appendix S2), and supplementary references (Appendix S3) are available online. The authors are solely responsible for the content and functionality of these materials. Queries (other than absence of the material) should be directed to the corresponding author. The code and data are available from www.github.com/jonrhodes/ConsPlanNetworks (https://doi.org/10.5281/zenodo.1409840).
