# Supplementary material for: Fundamental insights on when social network data are most critical for conservation planning
Source: Conserv Biol. 2020 Sep 5;34(6):1463–72. doi: 10.1111/cobi.13500 (PMC7754422; doi:10.1111/cobi.13500)
Supplement: Supplementary file 1 — Supplementary methods related to social network dynamics, species distributions, decision problem and optimization, value of social network information, small‐scale fishery (Appendix S1), supplementary figures and results (Appendix S2), and supplementary references (Appendix S3) are available online. The authors are solely responsible for the content and functionality of these materials. Queries (other than absence of the material) should be directed to the corresponding author. The code and data are available from www.github.com/jonrhodes/ConsPlanNetworks (https://doi.org/10.5281/zenodo.1409840). [file COBI-34-1463-s001.pdf]

Supporting Information

Title: Fundamental insights on when social network data are most critical for conservation planning

Appendix S1: Supplementary Methods

The code and data are stored in an online repository (www.github.com/jonrhodes/ConsPlanNetworks) and are available under the digital object identifier (DOI) 10.5281/zenodo.1409840. See the README file for instructions.

Social network dynamics

When the decision-maker does not intervene at a site/actor node, the transition probabilities from available to any other state are

$$\begin{aligned} P_{AD} &= p_d + (1 - p_d)\left(1 - (1 - u_d)^{n_d}\right) \\ P_{AR} &= 0 \\ P_{AA} &= 1 - P_{AD} \end{aligned} , \quad (1)$$

where P_{AD} is the transition probability from available to developed, P_{AR} is the transition probability from available to reserved, P_{AA} is the transition probability from available to available, p_d is the probability, independent of the network, that the actor associated with the site decides to develop the site, u_d is the probability that another actor who has developed their site influences the actor to develop, given they are connected in the network, and n_d is the number of actors who have already developed their sites that the actor is connected to. The probabilities of each actor influencing any other actor are assumed to be independent. This formulation assumes that there are two processes that determine whether an actor

develops their site or not. The first is the intrinsic propensity for the actor to develop their site, determined by the probability p_d. The second is the probability of being influenced to develop by other actors, who the actor is connected to, who have already developed their sites. If the actor decides, independently, not to develop their site (which occurs with probability 1 - p_d) then they can be subsequently influenced, through the network, to develop anyway with probability $1-(1-u_d)^{n_d}$. When there is no intervention by the decision-maker then we assume the transition probability to reserved is zero because there are no incentives present to do so.

When the decision-maker does intervene at a site/actor node, then the transition probabilities from available to any other state are

$$\begin{aligned} P_{AD} &= (1-P_{AR})\left(p_d + (1-p_d)\left(1-(1-u_d)^{n_d}\right)\right) \\ P_{AR} &= p_r + (1-p_r)\left(1-(1-u_r)^{n_r}\right) \\ P_{AA} &= 1 - P_{AD} - P_{AR} \end{aligned} \quad , \qquad (2)$$

where n_r is the number of actors who have already reserved their sites that the actor is connected to. Again, the probabilities of each actor influencing any other actor are assumed to be independent. In this case, we assume that the actor first decides whether to reserve or not and then decides whether to develop to not, if they have decided not to reserve. In Eq. (2) we again assume that there are two processes that determine whether an actor reserves their site or not. The first is the intrinsic propensity for the actor to reserve their site, determined by the probability p_r. The second is the probability of being influenced to reserve by other actors that the actor is connected to who have already reserved their sites. If the actor decides, independently, not to reserve their site (which occurs with probability 1 – p_r) then they can be

subsequently influenced, through the network, to reserve anyway with probability $1-(1-u_r)^{n_r}$. If the actor decides not to reserve their site (which occurs with probability 1 – P_{AR}), then they subsequently decide to develop their site with probability $p_d+(1-p_d)\left(1-(1-u_d)^{n_d}\right)$, following Eq. (2).

<u>Species distributions</u>

Patterns of species distributions varied with species nestedness and were generated statistically. As such, we do not model explicitly the underlying dynamics that lead to patterns of species nestedness, but rather just the pattern directly. Species distributions across sites were simulated based on four different parameters: the number of species in the species pool, S, the probability that a species occurs at a site, p, the level of nestedness, φ, and the number of sites, n. First, we defined the number of species at each site by drawing a binomial random variable for each site, with S trials, and probability of success p. Sites were then ranked from most species rich to least species rich and species identities were allocated to sites to match the number of species required at each site for a given level of nestedness. This was achieved by: (1) first allocating species' identities randomly, with equal probability, to the most species rich site, then, (2) iterating through the remaining sites in order of declining species richness and, at each site, allocating species j at site k, with the following probability:

$$w_{j,k}=\frac{\prod_{i=1}^{k-1}\varphi I_{i,j}+(1-\varphi)\left|I_{i,j}-1\right|}{\sum_{j=1}^{S}\prod_{i=1}^{k-1}\varphi I_{i,j}+(1-\varphi)\left|I_{i,j}-1\right|}, \quad (3)$$

where k is the site number from the second most ($k = 2$) to the least ($k = n$) species rich site, $I_{i,j} = 0$ if species j has not already been selected at site i, $I_{i,j} = 1$ if species j has already been selected at site i, and the denominator is a normalizing constant to ensure that all probabilities sum to one across species. If $w_{j,k} = 0$ for all species j at a site k, then all species are allocated randomly with equal probability. This formulation ensures that: (1) when $\varphi = 0.5$ species are distributed with a complete spatially random distribution across sites, (2) as $\varphi \to 1$ species become increasingly nested across sites (with low numbers of endemic species), and (3) as $\varphi \to 0$ species become increasingly un-nested across sites (with high numbers of endemic species). Fig. S1 illustrates how the probabilities of species selection vary with φ and the number of the species already selected in sites that are more species rich as the algorithm proceeds.

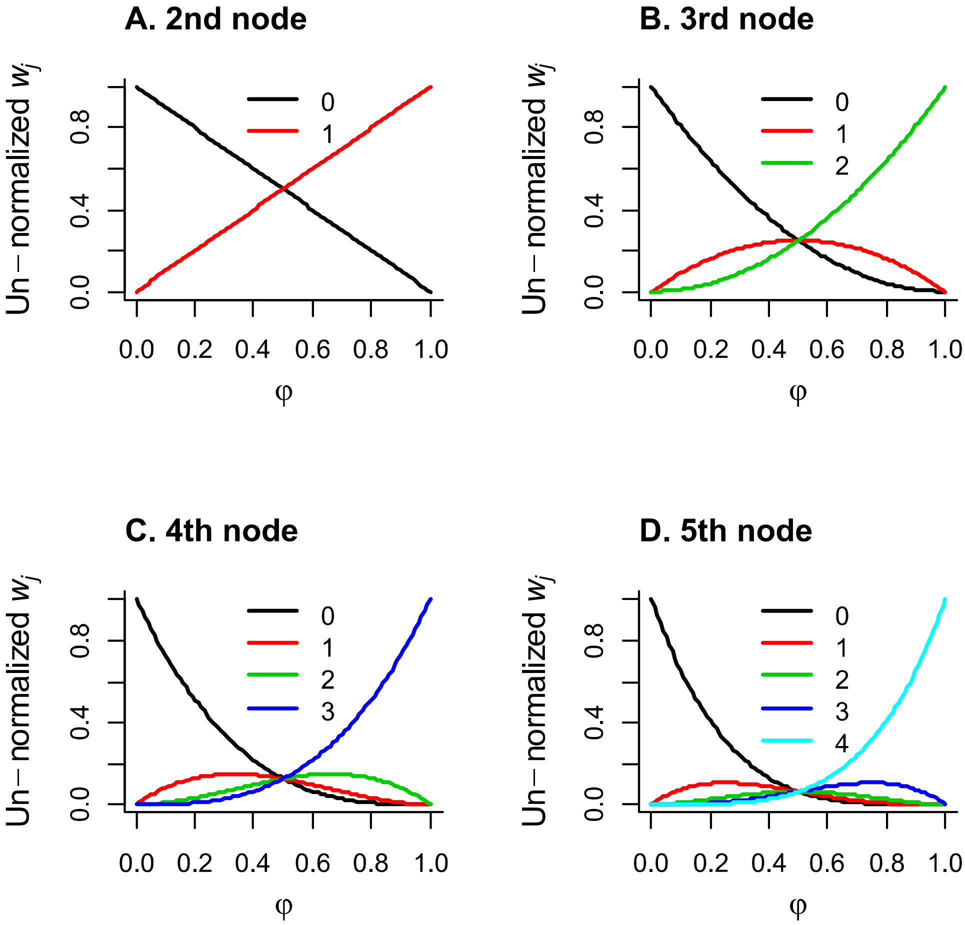

Figure S1. The probability of a species' selection at a site as a function of the site number (iteration), the nestedness, φ, and the number of sites the species has already been selected at. In all cases, when φ = 0.5, the probability is independent of the number of sites the species has already been selected at and so the distribution of species is completely spatially random. When φ < 0.5, the algorithm favors the selection of species that have few representations at other sites (resulting in an un-nested distribution, with many endemic species). When φ > 0.5, the algorithm favors the selection of species that already have representation at other sites (resulting in a nested distribution, with few endemic species).

Based on simulated species distributions, we found a strong negative correlation (ρ = -0.96, df = 8, $p = 1.6 \times 10^{-5}$) between φ and Brualdi and Sanderson's (1999) discrepancy index; a measure of the difference between a species pattern and a perfectly nested pattern (Fig. S2).

This demonstrates the ability of the algorithm to successfully generate species patterns with different levels of nestedness as defined by φ.

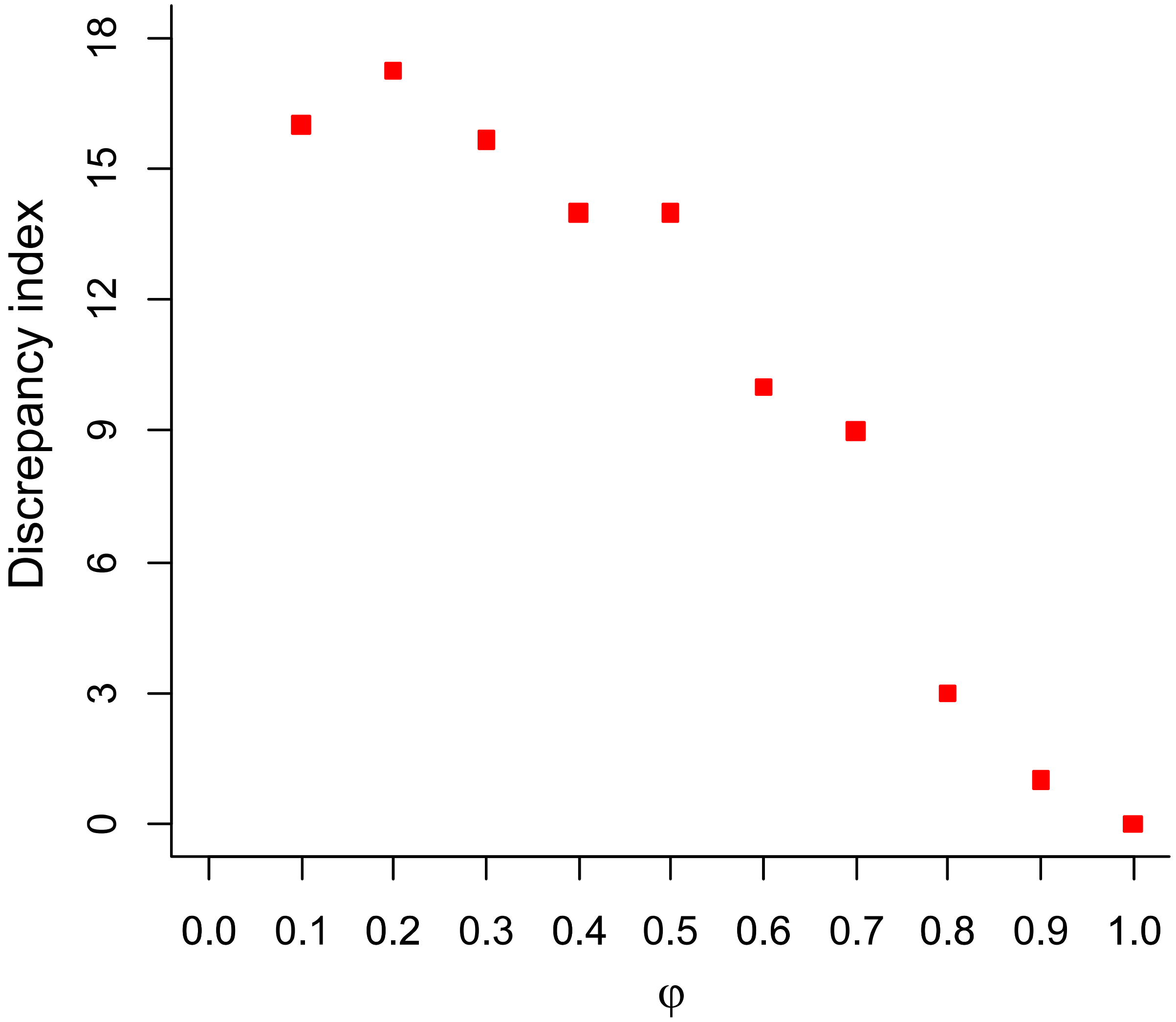

Figure S2. Relationship between φ and mean Brualdi and Sanderson's (1999) discrepancy index. Data based on simulations with n = 6, S = 20, p = 0.25, and 20 replicates for each value of φ.

All species distribution simulations were conducted in Matlab vR2016b and discrepancy index calculations were conducted in R v3.4.3 using the package 'vegan'.

Decision problem and optimization

We characterized system dynamics assuming a Markov process and discrete time steps based on the transition probabilities defined in Fig. 1 in the main text and above. We used artificial intelligence algorithms to solve the decision problem, formulated as an infinite-time horizon Markov Decision Process (MDP) (Hoey et al. 1999; Chadès et al. 2011) whereby a decision-maker undertaking actor-level interventions needs to decide which node to intervene at in at each time step to maximize the number of species in at least one reserve, time-discounted over an infinite time horizon. We assumed an arbitrary cost of 0.5 species for each intervention. This ensures that doing nothing is better than implementing an intervention when the expected outcomes are equivalent. However, we found that, when we ignored costs the overall conclusions did not change. We solved for the optimal state dependent strategy using value iteration for factored MDP (Hoey et al. 1999; Chadès et al. 2011), where a factored MDP explicitly accounts for the conditionally independent relationships between the action and state variables, resulting in computational gains. Factored MDPs also provide easy to interpret solutions from which essential mechanistic insights can be gained (Chadès et al. 2011). Below we describe the state transition probabilities and the optimization procedure in more detail.

We solved for the optimal state dependent intervention using a factored MDP (Hoey et al. 1999). Specifically, we defined $<S, A, \mathbf{P}, \mathbf{R}, \beta>$, where

> $S = S_1$ x … x S_n is a factored set of states representing all possible states of the network, with S_i taking values in {available, developed, reserved} for node i. The set S grows exponentially with the size of the number of nodes in the network so that $|S|=3^n$.

$A = \{0, \ldots, n\}$ is the set of possible actions and represents the possible nodes that the decision-maker can intervene at, assuming that they can only conduct one intervention per time step. Here $a \in A = 0$ represents the do nothing action and $a \in A > 0$ represents the node at which an intervention occurs. The set A grows linearly with the size of the number of nodes in the network so that $|A| = n + 1$.

P: S x A x S is the state transition matrix that defines the Markovian dynamics of the system. The probability of transitioning from one state $s_t \in S$ of the system at time t to the state at time $t+1$, $s_{t+1} \in S$, only depends on the current state s_t and the action implemented $a_t \in A$, $P\left(s_{t+1} \mid s_t, a_t\right)$.

R: S x A is the immediate reward matrix associated with the state and action implemented. In our case, the reward is the number of species in at least one reserve, minus the cost of engaging with an actor when an intervention is implemented, which we arbitrarily set at 0.5. Including a cost ensures that doing nothing is better than implementing an intervention when the expected outcomes are equivalent, however, we found that when we ignored costs, the overall conclusions did not change.

Finally, β is the discount factor. We set this value to 0.96.

The optimal state-dependent policy was then found by solving the following

$$V(s) = \max_{a \in A} \left\{ \mathbf{R}(s,a) + \beta \sum_{j \in S} \mathbf{P}(j \mid s,a) V(j) \right\}, \tag{4}$$

where $V(s)$ is the optimal reward in state *s*. This was solved using value iteration based on algebraic decision diagrams to take advantage of the conditional independence between the action and state variables in a factored MDP (Hoey et al. 1999). All optimizations were performed by using Matlab vR2016b to call the SPUDD: Stochastic Planning Using Decision Diagrams software (Hoey et al. 1999).

Value of social network information

To estimate the value of social network information, we generated five replicate species distributions for each combination of the following parameters: $S = 20$, $p = 0.25$, $\varphi = \{0.1,0.2,0.3,0.4,0.5,0.6,0.7,0.8,0.9,1.0\}$. Then, we generated social networks with all combinations of: $u_r = \{0.0,0.1,0.2,0.3,0.4,0.5,0.6,0.7,0.8,0.9,1.0\}$ and $u_d = \{0.0,0.1,0.2,0.3,0.4,0.5,0.6,0.7,0.8,0.9,1.0\}$, with $p_r = 0.2$ and $p_d = 0.2$ (see above and Table S1 below for definitions of parameters). This generated 1,210 species distribution/social network parameter combinations for each network motif, with five species distribution replicates per combination (a total of 6,050 simulated instances per motif).

Table S1. Definition of parameters and values considered in the simulations for each motif.

**Parameter**	**Description**	**Values considered for each motif**
S	Number of species in the species pool	20
p	Probability that a species occurs at a site	0.25
φ	Level of species nestedness ($0 < \varphi \leq 1$, with $\varphi = 1$ being the most nested)	0.1,0.2,0.3,0.4,0.5,0.6,0.7,0.8,0.9,1.0
p_d	Probability, independent of the network, that the actor associated with a site decides to develop the site	0.2
u_d	Probability that an actor, who has already developed their site, influences an actor to develop, given they are connected in the network	0.0,0.1,0.2,0.3,0.4,0.5,0.6,0.7,0.8,0.9,1.0
p_r	Probability, independent of the network, that the actor associated with a site decides to reserve the site	0.2
u_r	Probability that an actor, who has already reserved their site, influences an actor to reserve, given they are connected in the network	0.0,0.1,0.2,0.3,0.4,0.5,0.6,0.7,0.8,0.9,1.0
β	Discount factor	0.96

For each species distribution/social network parameter combination and species distribution replicate we found the optimal policy assuming: (1) full information about the social network and the species distributions and, (2) full information about the species distributions, but

ignoring social network structure (i.e., assuming $u_r = 0$ and $u_d = 0$). Then, we simulated each optimal policy over a period of 150 time-steps (assuming all nodes are initially available), with the underlying dynamics described by the model that accounts for the full social network dynamics. This was replicated 1,000 times for each species distribution/social network combination and species distribution replicate. Based on these simulations, the value of information about the social network was estimated as the difference in the natural log of the expected optimal outcome (number of species in reserves minus cost) when information on the social network is used to find the optimal policy and when it is ignored. We used the difference in the natural log of the expected outcome so that the value of information measure is approximately the proportional improvement in conservation outcomes, rather than the absolute improvement.

Generalized linear models were then used to quantify the effect of the parameters on the value of social network information. To do this, for each network structure, we fitted a generalized linear mixed-effects model to the simulated log differences between the performance (i.e., the total reward [number of species in at least one reserve, minus implementation costs]) when making optimal decisions with and without network information. This regression took the following form

$$\begin{aligned} D_{i,j} &\sim \mathrm{Normal}\left(\mu_{i,j}, \sigma^2\right) \\ L_{i,j} &\sim \mathrm{Normal}\left(D_{i,j}, \phi_{i,j}^2\right), \end{aligned} \tag{5}$$

where $D_{i,j}$ is the true difference in log-rewards for network parameter combination *j* (i.e., the parameter combinations that define the network structure and dynamics) associated with species distribution replicate *i*, $\mu_{i,j}$ is the expected difference in log-rewards for network

parameter combination j associated with species distribution replicate i, σ^2 is the variance of the difference in log-rewards, $L_{i,j}$ is the simulated difference in log-rewards for network parameter combination j associated with species distribution replicate i, and $\phi^2_{i,j}$ is the variance of the observed difference in the log-rewards for network parameter combination j associated with species distribution replicate i (estimated from the 1,000 evaluation replicates for each network parameter combination j associated with species distribution replicate i).

We constructed the model so that $\mu_{i,j}$ was a function of the model parameters and accounted for the correlated/nested structure of the simulated data (i.e., network parameter combinations nested within species distribution replicates). Specifically, we set

$$\mu_{i,j} = a + bNST_i + c_i PIR_{i,j} + d_i PID_{i,j} + \eta_i, \quad (6)$$

where NST_i is the species nestedness, φ, for species distribution replicate i, $PIR_{i,j}$ is the probability of reserve influence, u_r, for network parameter combination j associated with species distribution replicate i, and $PID_{i,j}$ is the probability of development influence, u_d, for network parameter combination j associated with species distribution replicate i. Here a, b, c_i, and d_i are regression coefficients, and η_i is a normally-distributed random-effect for species distribution replicate i. Note that because the network parameter combinations are nested within the species distribution replicates, the coefficients for $PIR_{i,j}$ and $PID_{i,j}$ vary across species distribution replicates. To quantify potential interactions between the species distribution parameters and the social network parameters, we further defined c_i and d_i as functions of the species distribution parameters

$$c_i = \alpha_d + \beta_d SN_i + \gamma_d OC_i + \chi_d NST_i + \kappa_i$$
$$d_i = \alpha_e + \beta_e SN_i + \gamma_e OC_i + \chi_e NST_i + \varepsilon_i \quad , \qquad (7)$$

Where SN_i is the number of species in the species pool for species distribution replicate *i*, OC_i is the probability a species occupies a site for species distribution replicate *i*, $\alpha_d, \beta_d, \gamma_d, \chi_d$ and $\alpha_e, \beta_e, \gamma_e, \chi_e$ are regression coefficients, and κ_i and ε_i are normally-distributed random-effects for species distribution replicate *i*.

Models were fit to the simulated data using Markov Chain Monte Carlo (MCMC) in JAGS v4.3.0 using the package 'runjags' in R v3.4.3 and essentially uninformative prior distributions. Convergence was assessed using the Gelman and Rubin convergence statistic (R-hat) (Gelman & Rubin 1992).

Small-scale fishery

The small scale fishery is located south of Mombasa, Kenya and we used network and species data from Bodin et al. (2014) and Crona et al. (2006). We assumed that the information exchange/knowledge transfer links described by the social network influence the propensity of groups of fishers to behave similarly to each other, as we did for the simulations. However, since we also had information on relative link strengths, we allowed influence probabilities to vary among network links. The absolute probability of influence is not known in this system, so instead we first scaled link strengths to be less than one by dividing all link strengths by the maximum link strength and then multiplied these values by a maximum permitted influence probability (equivalent to the u_d and u_r parameters), which we varied systematically between zero and one.

Each of the fisher groups target different species, hence the species associated with each group, at their fishing sites differ, but there is some overlap in the species that they target. We made the assumption that each fisher group starts by fishing sustainably (i.e., prior to the fishery becoming unsustainable) and, in this state, the species associated with each node persist in the short-term (equivalent to the 'available' state in the simulations). However, a fisher group can decide to fish unsustainably, due to demand, market forces, or commercialization of the fishery (as reflected by the long-term trajectory of the fishery) and, in this case, we assume that the species associated with the node no longer persist in the long-term (equivalent to the 'developed' state in the simulations). We set the probability of adopting long-term unsustainable fishing practices to 0.2 (equivalent to p_d in our simulations). We also assumed that a decision-maker can intervene at a fisher group to incentivize the group to not transition to the long-term unsustainable state, in which case the species associated with the node persist in the long-term (equivalent to the 'reserved' state in our simulations). Finally, we assumed that the probability of adopting long-term sustainable fishing practices, given an intervention, was 0.2 (equivalent to p_r in our simulations). Note that, as for the simulations, we assumed an infinite time horizon and so only species in nodes with long-term sustainable harvesting (the 'reserved' state) contribute to the conservation objective.

We identified optimal state-dependent actor-level interventions to incentivize sustainable fishing practices for the different fisher groups/sites so as to maximize the number of species in sites with long-term sustainable harvesting (i.e., reserved), minus the costs of intervention (assumed to be 0.5, as in the simulations). We then estimated the value of social network information for each parameter combination and assessed how knowing information about the network influences the optimal sequence of interventions.

Appendix S2: Supplementary Figures and Results

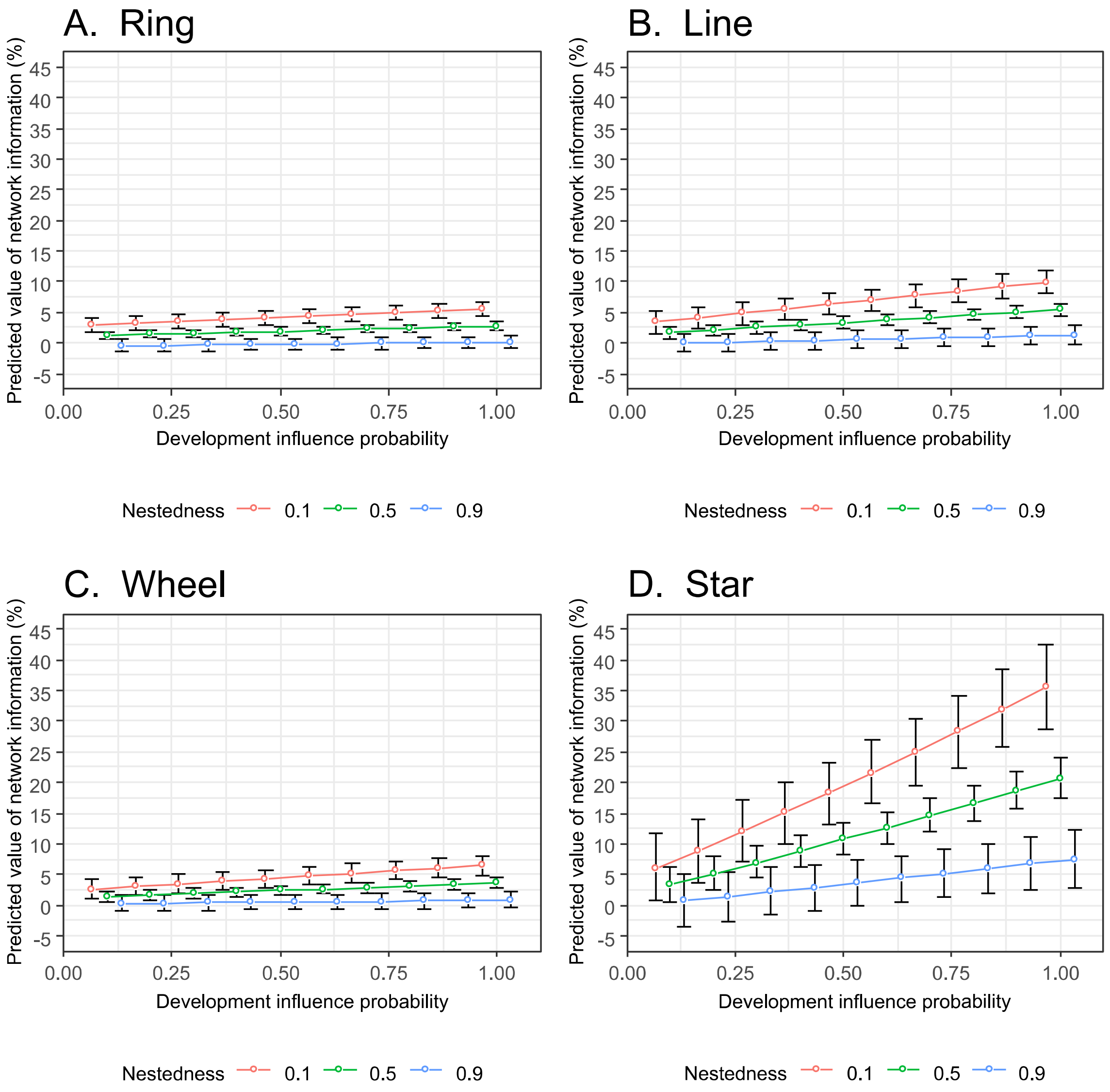

Figure S3. Estimated value of social network information (and 95% credible intervals) for four of the motifs with different values of development influence probability and species' nestedness. The value of network information shown is approximately the percentage improvement in conservation benefits achieved by including social network information in the prioritization. These values are predictions from the generalized linear regression model, with other parameters set at their mean values. The results for the ring-star network are not shown because they are simply intermediate between the results for the ring and the star networks.

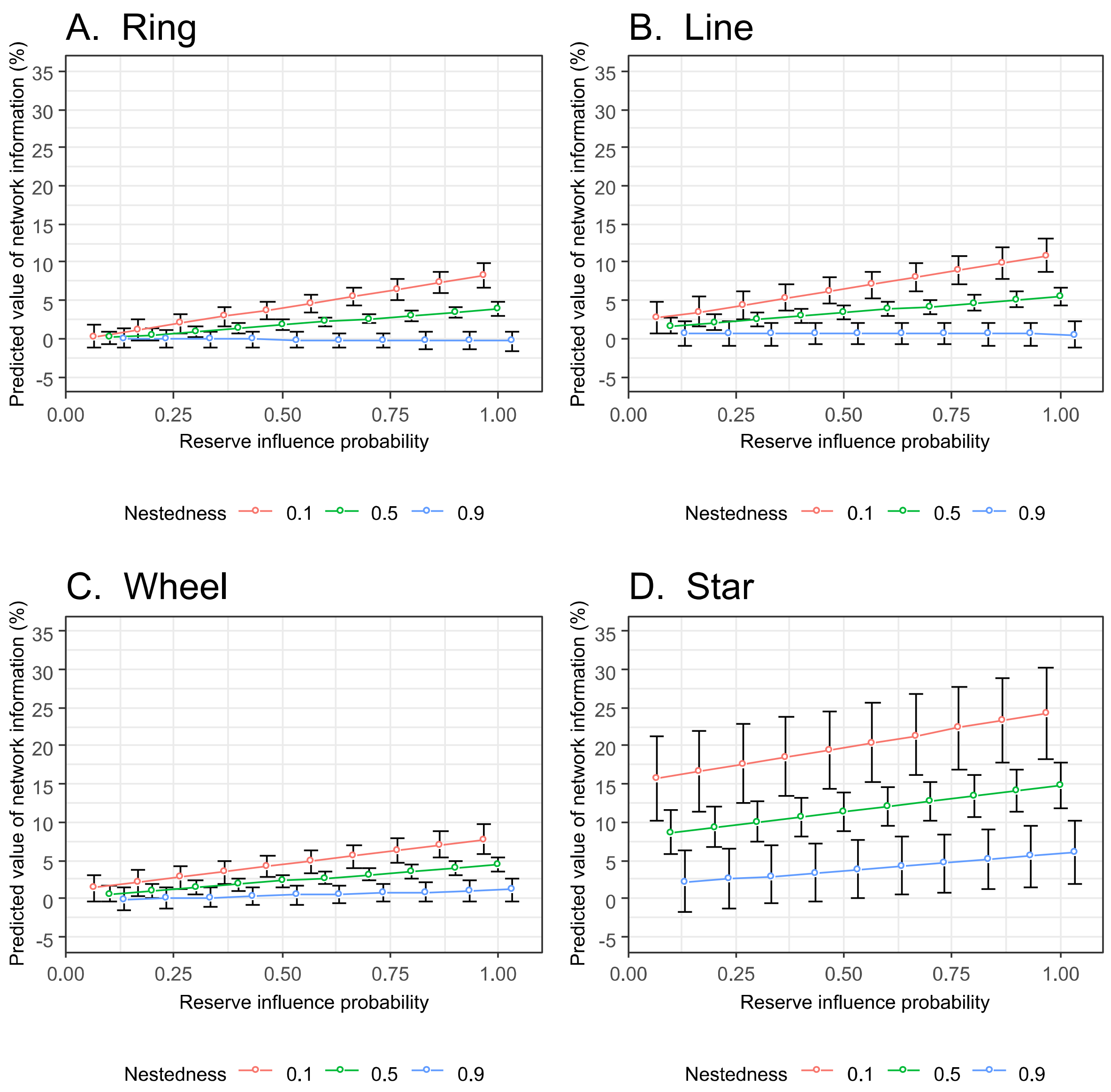

Figure S4. Estimated value of social network information (and 95% credible intervals) for four of the motifs with different values of reserve influence probability and species' nestedness. The value of network information shown is approximately the percentage improvement in conservation benefits achieved by including social network information in the prioritization. These values are predictions from the generalized linear regression model, with other parameters set at their mean values. The results for the ring-star network are not shown because they are simply intermediate between the results for the ring and the star networks.

Notes on Figures S3 and S4 and the value of information estimates

In Fig. S3 and Fig. S4 some predictions indicate some slightly negative value of information estimates. Although, in deterministic systems, value of information should never be less than zero, in our stochastic system negative estimates may be possible simply due to stochastic variation. However, in some cases, where species nestedness is high and network centrality is very low (e.g., the ring network), negative values of information were obtained beyond what can be explained purely by stochastic variation in the simulations. We hypothesize that this likely relates to the way we have approximated value of information by first finding the optimal policy to maximize the expected reward, and then applying the optimal policy to stochastic rewards in the simulations and then taking the expectation of those. Since the value of a function applied to the expected rewards (i.e., finding the optimal policy) is not equal to the value of a function applied to the stochastic rewards and then taking the expectation (i.e., applying the policy to the simulation) for a nonlinear function, this could lead to negative values of information estimates. Although this effect may have influenced the absolute estimates of value of information slightly, it is, however, unlikely to have affected the overall patterns we discovered with respect to network structure and species nestedness.

Appendix S3: Supplementary Literature Cited

Bodin O, Crona B, Thyresson M, Golz AL, Tengö M. 2014. Conservation success as a function of good alignment of social and ecological structures and processes. Conservation Biology **28**:1371-1379.

Brualdi RA, Sanderson JG. 1999. Nested species subsets, gaps, and discrepancy. Oecologia **119**:256-264.

Chadès I, Martin TG, Nicol S, Burgman MA, Possingham HP, Buckley YM. 2011. General rules for managing and surveying networks of pests, diseases, and endangered

species. Proceedings of the National Academy of Sciences of the United States of America **108**:8323-8328.
Crona B, Bodin O. 2006. What you know is who you know? Communication patterns among resource users as a prerequisite for co-management. Ecology and Society **11**.
Gelman A, Rubin DB. 1992. Inference from iterative simulation using multiple sequences. Statistical Science **7**:457-511.
Hoey J, St-Aubin R, Hu A, Boutilier C. 1999. SPUDD: stochastic planning using decision diagrams. Pages 279-288 in Laskey K, and Prade H, editors. Fifteenth Conference on Uncertainty in Artificial Intelligence. Morgan Kaufmann, Stockholm, Sweden.
